# Clinical impact of molecular diagnosis in dog allergy

**DOI:** 10.1186/2045-7022-4-S2-P52

**Published:** 2014-03-17

**Authors:** Silvia Uriarte Obando, Joaquín Sastre Domínguez

**Affiliations:** 1 Fundación Jiménez Díaz, Allergy Department, Madrid, Spain

## Background

Allergy to dog is a frequent cause of rhinitis or asthma. The prevalence of sensitization to different dog allergens is not well known.

## Methods

We select 159 sensitized patients allergic to dog. Specific IgE measurement to dog allergens Can f 1, Can f 2, Can f 3 and Can f5 was performed by ImmunoCAP® and/or microarray ISAC® (ThermoFisher Scientific, Sweden), a value > 0,35 kU/L or >0,3 ISU was considered as positive, respectively. Association of Specific IgE measurements was done with presence and type of rhinitis or asthma.

## Results

79% of patients had specific IgE to Can f1, 19% to Can f2, 12% to Can f3, and 35% to Can f5. 44% were monosensitized to Can f 1, 19.5% to Can f5 and 0.6% to Can f3. Can f1 was associated with persistent rhinitis (p 0.01), Can f3 with severity of rhinitis and asthma (p <0.01, p 0.01, respectively), and Can f5 to both persistence and severity of rhinitis (p 0.02, p >0.001, respectively). Sensitization to several allergens in patients (1, 2, 3 or 4) was associated with persistent asthma or rhinitis (p 0.04, p 0.01, respectively), and with moderate severity (p 0.03). Direct contact with dogs was associated with both, persistency and severity of rhinitis (p 0.02, p 0.03, respectively). The wheal diameters of skin test with commercial extract of dog were smaller in patients monosensitized to Can f 5.

## Conclusion

Different patterns of sensitization to dog allergens that are commercially available, in patients with dog allergy can help us to predict the severity and persistence of symptoms as well as sensitization to a higher number of dog allergens. Interestingly, a high prevalence of monosensitization to Can f 5 was demonstrated. Can f 5 is an arginine esterase or prostatic kallikrein that is found only in male dogs. This finding could be of clinical interest because may explain differences in development of symptoms when exposed to male or female dogs. Further studies are necessary to establish if different patterns of sensitization to dog allergens may have clinical consequences and response to immunotherapy.

